# Dosimetric comparison of three‐dimensional conformal radiotherapy and intensity‐modulated radiotherapy for left‐sided chest wall and lymphatic irradiation

**DOI:** 10.1002/acm2.12757

**Published:** 2019-11-03

**Authors:** Huseyin Kivanc, Melis Gultekin, Murat Gurkaynak, Gokhan Ozyigit, Ferah Yildiz

**Affiliations:** ^1^ Department of Radiation Oncology School of Medicine Hacettepe University Ankara Turkey

**Keywords:** breast cancer, chest wall irradiation, intensity‐modulated radiotherapy, lymphatic irradiation, three‐dimensional conformal radiotherapy

## Abstract

**Introduction:**

The aim of this study was to compare five different techniques for chest wall (CW) and lymphatic irradiation in patients with left‐sided breast carcinoma.

**Methods:**

Three‐dimensional conformal radiotherapy (3DCRT), forward‐planned intensity‐modulated radiotherapy (FP‐IMRT), inverse‐planned IMRT (IP‐IMRT; 7‐ or 9‐field), and hybrid IP‐/FP‐IMRT were compared in 10 patients. Clinical target volume (CTV) included CW and internal mammary (IM), supraclavicular (SC), and axillary nodes. Planning target volumes (PTVs), CTVs, and organs at risks (OARs) doses were analyzed with dose–volume histograms (DVHs).

**Results:**

No differences could be observed among the techniques for doses received by 95% of the volume (D95%) of lymphatics. However, the FP‐IMRT resulted in a significantly lower D95% dose to the CW‐PTV compared to other techniques (*P* = 0.002). The 9‐field IP‐IMRT achieved the lowest volumes receiving higher doses (hotspots). Both IP‐IMRT techniques provided similar mean doses (Dmean) for the left lung which were smaller than the other techniques. There was no difference between the techniques for maximum dose (Dmax) of right breast. However, FP‐IMRT resulted in lower Dmean and volume of right breast receiving at least 5 Gy doses compared to other techniques.

**Conclusion:**

The dose homogeneity in CW‐CTV was better using IMRT techniques compared to 3DCRT. Especially 9‐field IP‐IMRT provided a more homogeneous dose distribution in IM and axillary CTVs. Moreover, the OARs volumes receiving low radiation doses were larger with IP‐IMRT technique, while volumes receiving high radiation doses were larger with FP‐IMRT technique. Hybrid IMRT plans were found to have the advantages of both FP‐ and IP‐IMRT techniques.

## 1 INTRODUCTION

Radiotherapy has a major role in the management of breast cancer that reduces the risk of loco‐regional recurrences and improves overall survival both for early stage breast cancer after breast‐conserving surgery and locally advanced disease after mastectomy.^1^, ^2^ However, irradiation of the chest wall (CW) and regional lymph nodes is one of the most difficult and challenging techniques in radiation oncology. Coverage of CW and regional lymphatics including supraclavicular (SC), axillary, and internal mammary (IM) lymph nodes needs special attention to the doses of lungs, heart, and the opposite breast tissue.

Treatment planning is most commonly implemented using a three‐dimensional (3D) conformal technique. Arthur et al. showed improved target coverage and reduction in normal tissue doses with partially wide tangent fields when compared with widened tangents or a 5‐field technique using a photon/electron mix.^3^ However, when treating left‐sided breast cancer, the concave shape of the CW results in suboptimal dose homogeneity, target coverage, and unavoidable irradiation to portions of the underlying lung and heart with 3D conformal radiotherapy (3DCRT).

Intensity‐modulated radiotherapy (IMRT) is an advanced form of 3DCRT that has been shown to improve target coverage and dose homogeneity compared to 3DCRT.^4^ In addition, IMRT can decrease the dose to heart and lung. Forward‐planned IMRT (FP‐IMRT) with field‐in‐field (FINF) technique allows more homogenous dose distribution with reduced lung and heart doses. In recent years, inverse‐planned IMRT (IP‐IMRT) has been shown to improve breast and regional node coverage while decreasing dose to lung, heart, and contralateral breast tissue.^5^ Patients with larger breasts who frequently have large‐dose inhomogeneities are most likely to benefit from IMRT. Recent trials have demonstrated that the use of the simplified IMRT technique resulted in improved dose homogeneity in the breast while resulting in lower skin and soft tissue toxicity than two‐dimensional treatment planning.^6^, ^7^, ^8^ IMRT technique can also be an alternative for left‐sided breast cancers to decrease cardiac dose.^4^, ^9^, ^10^, ^11^, ^12^

Although there are several IMRT techniques and many dosimetric and planning studies in the literature showed better dose homogeneity with decreased organs at risk (OAR) doses, the role and consequence of IP‐IMRT in breast carcinoma need to be defined. The primary aim of this study was to compare five different techniques including 3DCRT, FP‐IMRT, IP‐IMRT, and hybrid IP‐IMRT/FP‐IMRT for CW and full lymphatic irradiation with respect to target volumes and doses to critical structures in patients with left‐sided breast carcinoma. This study also aimed to define the ideal treatment plan according to the treatment planning system (TPS) and the dosimetric analysis.

## 2 MATERIALS AND METHODS

### 2.1 Patient selection, positioning, and computed tomography scans

The computed tomography (CT) scans of 10 patients with left‐sided breast carcinoma were used for this dosimetric study. All patients were positioned supine with left arm raised above the head on a breast board. Patients were scanned from the level of the mandibula through to the upper abdomen, including left and right lungs, with a 2.5‐mm slice thickness on a GE™ HiSpeed NX/i (GE™ HiSpeed NX/i; GE Medical Systems, Little Chalfont, UK) CT system.

### 2.2 Target volumes and critical structures

All clinical target volumes (CTVs) were contoured according to the RTOG contouring atlas by a single radiation oncologist using Varian Eclipse Operation version 8.9 treatment planning software (Eclipse treatment planning system; Varian Medical Systems, Palo Alto, CA).^13^ A planning target volume (PTV) margin of 5 mm was added to all CTVs in order to overcome the set‐up uncertainty and internal organ motion. A PTVeval was also produced to pull back the PTV 1 mm below the skin surface for CW and 5 mm for lymphatics and exclude left lung from posterior rib surface. Normal tissues including the heart, lung, brachial plexus, spinal cord, thyroid, right breast, esophagus, and head of humerus were contoured as OARs. In addition, a structure “external” encompassed all normal tissues not otherwise specified (such as the arm, posterior thorax, and soft tissues) by substracting the volumes of the targets and specified normal tissues from the external surface. A fractionation schedule of 50 Gy in 2 Gy per fractions was used. The details of dose prescription are given in Table 1.

**Table 1:** Dose‐volume constraints for the targets and critical structures

All PTVs	D_95%_ ≥4500 cGy			D_2%_ ≤5350 cGy				D_max_ ≤5500 cGy	
All CTVs	D_98%_ ≥4750 cGy			D_2%_ ≤5350 cGy				D_max_ ≤5500 cGy	
Heart	*V_5Gy_*	*V_10Gy_*			*V_20Gy_*	*V_30Gy_*			*Mean Dose*
	≤%50	≤%30			≤%10	≤%3			≤5 Gy
Left Lung	*V_20Gy_*								
	≤20%								
Contralateral Breast	*V_5Gy_*		*Mean Dose*				*Maximum Dose*		
	≤5%		250 cGy				≤3500 cGy		
Esophagus	D_max_ ≤3000 cGy								
Thyroid	V_50Gy_ ≤50%								
Humeral Head	D_max_ ≤5000 cGy								
Spinal Cord	D_max_ ≤4500 cGy								
Brachial Plexus	D_max_ ≤5000 cGy								

Abbreviations: CTV = clinical target volume; D_95%_ = lowest dose received by at least 95% of the volume, for example, D_98%_ is the lowest dose received by at least 98% of the volume, etc.; D_max_ = Maximum dose; PTV = planning target volume; V_5Gy_ = percentage of the volume receiving 5 Gy or more, for example, V_10Gy_ is the percentage of the volume receiving 10 Gy or more, etc.

### 2.3 3DCRT, FP‐IMRT, IP‐IMRT, and hybrid IP‐IMRT/FP‐IMRT techniques

**Figure 1:**
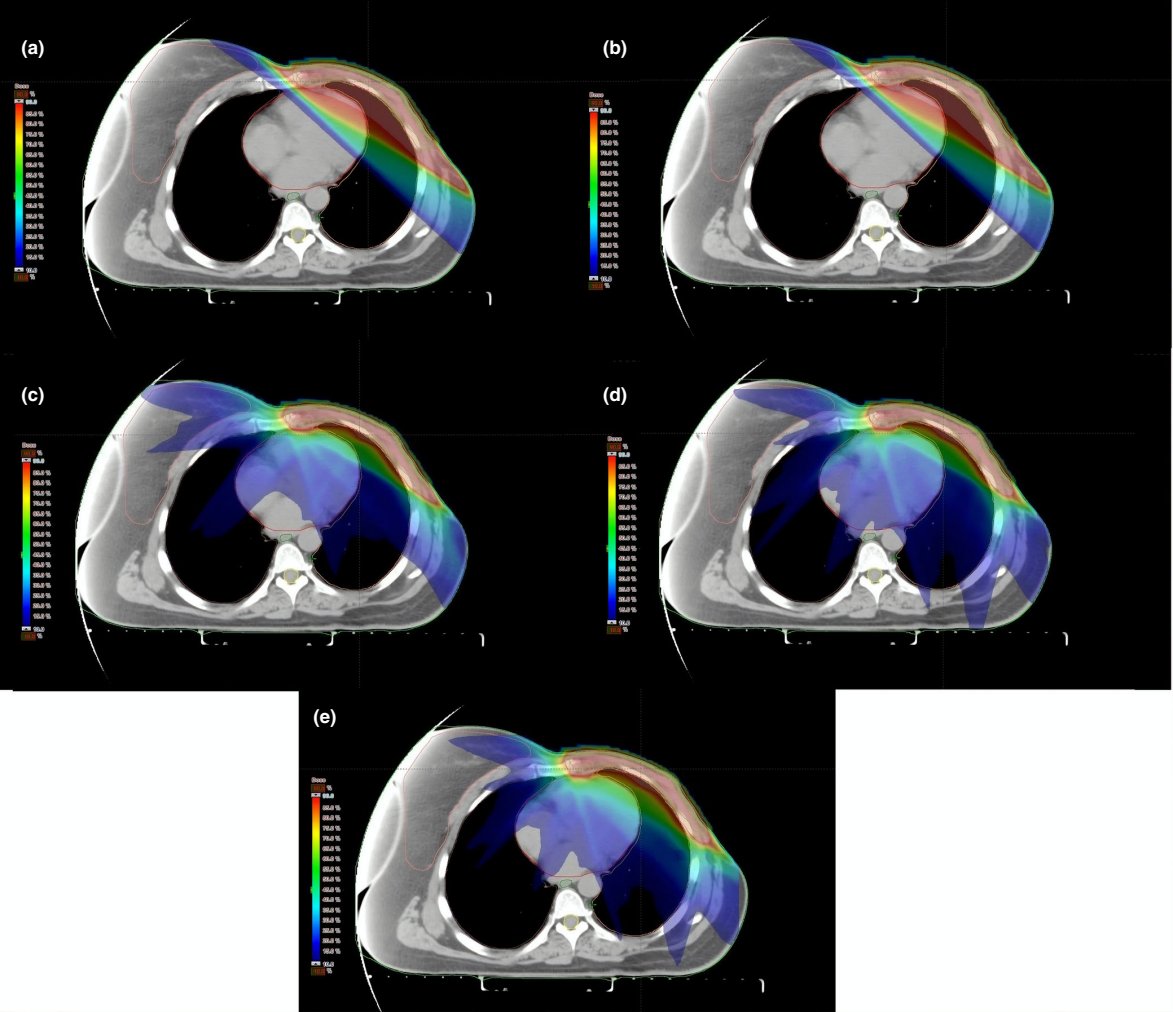
Impact of different treatment techniques on dose distribution in an axial image of same patient (a) Three‐dimensional conformal radiotherapy, (b) forward‐planned intensity modulated radiotherapy (IMRT), (c) 7‐field inverse‐planned IMRT, (d) 9‐field IP‐IMRT, (e) hybrid IMRT.

For each patient, 3DCRT, FP‐IMRT, IP‐IMRT (7‐ or 9‐field), and hybrid IP‐IMRT/FP‐IMRT plans were created. All dose–volume histograms (DVHs) obtained from different treatment techniques were evaluated for target volumes and critical structures. For 3DCRT, partially wide tangential fields without wedges were used in order to cover the CW and IM lymphatics and anterior and posterior oblique fields were used to cover the SC and axillary lymphatics. 6 MV photon beams were used for tangential fields and anterior SC‐axillary fields, whereas 18 MV photon beams were used for posterior SC‐axillary fields. Beam shaping was accomplished with multileaf collimators (MLC) to shield the heart and lungs as needed. In the FP‐IMRT technique, FINF technique was used with the same isocenter as the 3DCRT plans. Five to 10 percent of dose was given with additional fields with manually created apertures to block specific hotspots or lung/heart tissues to improve dose homogeneity. For the IP‐IMRT technique, we used seven fields (gantry angles: 300°, 315°, 340°, 35°, 80°, 100°, 120°) or nine fields (gantry angles: 300°, 315°, 340°, 15°, 35°, 55°, 80°, 100°, 120°) around each patient in the axial plane [Figs. 1(c) and 1(d)]. This technique used optimization algorithms to create fluence maps to shape dose distributions. All plans in IP‐IMRT were done using 6 MV photon beams. The most appropriate beam angles were chosen on the basis of patient anatomy and the position of the target volumes and surrounding critical structures. The hybrid IP‐IMRT/FP‐IMRT plans consisted of a 9‐field IMRT and FP‐IMRT plan with energy of 6 MV [Fig. 1(e)]. In this hybrid technique, all the plans first made using either 40%–60%, 20%–80%, 60%–40%, or 80%–20% combinations of FP‐IMRT and IP‐IMRT. After evaluating the DVHs of combination plans, it was decided that the best combination could be achieved with the combination of IP‐IMRT as 80% and FP‐IMRT as 20% of the treatment fractions.

### 2.4 Dosimetric measurements

An individual simulation was done using an Alderson rando^R^ phantom (Alderson rando phantom; Phantom Laboratory, Salem, New York, USA) for each technique. Field borders were defined, and CT markers were placed to delineate margins. The CT scans of Alderson rando^R^ phantom were transferred to the TPS, and treatments were planned with five different techniques. After determination of treatment fields in rando phantom, thermoluminescent dosimeters (TLDs) were placed at certain depths which were thought to represent the SC, axillary, and IM lymphatics. Additional TLDs were put on several points that were thought to represent the right breast, CW, lung, and heart. In order to determine surface doses, Gafchromic^TM^ EBT (Gafcromic EBT films; International Specialty Products, New Jersey, USA) dosimetry films were placed on the CW and right breast with 0.5 mm tissue equivalent bolus material or without bolus. In this way, two different measurements were taken for each plan. TLD and Gafchromic^TM^ EBT dosimetry films were calibrated before treatment.

### 2.5 Statistical analysis

All statistical analysis was performed using SPSS, version 18.0 (SPSS, Illinois, Chicago, USA). Plan evaluation parameters for each structure and deviation from dose constraints were calculated for each plan. Fifty different DVHs were calculated for all target volumes, including the CW, SC, axillary, and IM chain and OAR. Dose homogeneity was evaluated using the minimal dose (Dmin), maximal dose (Dmax), mean doses (Dmean), D95% (doses received by the 95% of the target volume), D2% (dose to 2% of the volume), D98% (dose to 98% of the volume), and standard deviations were defined. Homogeneity Index (HI) was also calculated: HI = (D_2_ − D_98_/D_prescribed_) × 100. Friedman test was used for comparison. A *P* < 0.05 was considered significant. Also, the mean values obtained from the TLD and Gafchromic^TM^ EBT dosimetry films and standard deviations were compared to the doses of the same points in the TPS. In addition, all the plans were compared for monitor units (MUs) required for treatment delivery.

## 3 RESULTS

### 3.1 Results of the dose–volume histograms

Table 2 shows the dose–volume indices for target volumes and critical structures obtained from 50 treatment plans for all ten patients. No differences could be observed among the techniques for D95% doses of IM, SC, and axillary lymphatics. However, the FP‐IMRT resulted in a significantly lower D95% dose to the CW‐PTV compared to other techniques (*P* = 0.002). 3DCRT provided significantly higher D2%, Dmax, and Dmean doses to CW‐CTV compared to the other techniques. IMRT techniques resulted in more homogeneous dose distribution in CW‐CTV (HI_7field_ = 12.9 and HI_9field_ = 12) compared to 3DCRT techniques (HI = 18.2) without any significant difference between IMRT techniques.

**Table 2:** Dose–volume indices for target volumes and critical structures.

	3DCRT	FP‐IMRT	7‐field IP‐IMRT	9‐field IP‐IMRT	Hybrid IMRT	
Structure	Mean estimate (SD)	Mean estimate (SD)	Mean estimate (SD)	Mean estimate (SD)	Mean estimate (SD)	*P* value
Targets						
Chest wall PTV						
D_95%_ (cGy)	4638.1 (93.4)	*4607.9 (82)	4660.5 (54)	4679.4 (59.1)	4708.2 (56)	0.002
Chest wall CTV						
Mean dose (cGy)	*5060.7 (85.4)	4976.9 (84.3)	4986.4 (56.9)	5052 (43.7)	5037 (40.6)	0.007
D_98%_ (cGy)	4765.3 (82.6)	4756.3 (64.6)	4757.2 (11.6)	4780 (59.3)	4772 (56.4)	0.086
D_2%_ (cGy)	*5673.3 (54.6)	5475.3 (45.6)	5404 (97.3)	5380 (25.2)	5423 (47.5)	0.004
D_max_ (cGy)	*5834 (316)	5497 (54.2)	5459.6 (94.6)	5412 (106.6)	5452 (50.6)	<0.001
Internal mammary PTV						
D_95%_ (cGy)	4634.6 (212.6)	4609.9 (210)	4625.2 (36.7)	4632.5 (37.9)	4678 (74.1)	0.318
Internal mammary CTV						
Mean dose (cGy)	4990.2 (89.3)	4927.7 (195)	4971.9 (83.4)	5014.8 (50.5)	4988.5 (57.4)	0.086
D_98%_ (cGy)	4755.2 (202.5)	4736.4 (232.5)	4750.8 (161.3)	4749.5 (179.2)	4770 (150.3)	0.392
D_2%_ (cGy)	5250.4 (185.1)	5148.6 (125.1)	5186.7 (84.3)	*5251.9 (73.6)	5187.1 (67.6)	0.040
D_max_ (cGy)	5345.4 (158.1)	5280.2 (65.5)	5268.3 (94.8)	*5353.4 (83.7)	5268.3 (77.8)	0.026
Supraclavicular PTV						
D_95%_ (cGy)	4701.7 (57.4)	4689.9 (129.1)	4656 (47.6)	4682.4 (55.2)	4686.6 (57.4)	0.392
Supraclavicular CTV						
Mean dose (cGy)	5010 (112.4)	5008.8 (102)	4989.9 (50.8)	4998 (62.1)	5003.7 (71.2)	0.072
D_98%_ (cGy)	4789.3 (138)	4778.6 (134)	4728.9 (108)	4749.8 (123)	4786.9 (117)	0.086
D_2%_ (cGy)	5390.3 (121)	5370.3 (116)	5359.4 (45.6)	5387.3 (45)	5343.4 (84.9)	0.182
D_max_ (cGy)	5495 (75.9)	5430.8 (111.9)	5469.4 (101.3)	5477.3 (45)	5423.9 (89)	0.228
Axillary PTV						
D_95%_ (cGy)	4677.3 (79.3)	4641 (73.3)	4593.1 (58.9)	4674.9 (44.8)	4633.1 (54.1)	0.696
Axillary CTV						
Mean dose (cGy)	4988.7 (82.4)	4980.2 (78.2)	4973.6 (87.9)	4928.4 (53.5)	4983.3 (43.8)	0.620
D_98%_ (cGy)	4748.3 (88.2)	4735.6 (56.2)	4720.7 (102)	4740.8 (94.6)	4786.9 (85.9)	0.088
D_2%_ (cGy)	*5403.3 (45.6)	5350.3 (75.3)	5335.4 (45.7)	5263.6 (85.3)	5300.4 (95.3)	0.003
D_max_ (cGy)	*5504 (75.9)	5475.3 (43.8)	5445.9 (78.9)	5468.3 (82.7)	5483.9 (78.7)	0.002
Normal tissues						
Heart						
Mean dose (cGy)	880.6 (399.8)	877.9 (399)	958.4 (166.2)	966.4 (161.2)	934.9 (197)	0.631
V_5_ (%)	27.5 (13.7)	*27.4 (13.6)	86.3 (7.3)	89.6 (6.7)	83.3 (8.7)	<0.001
V_10_ (%)	21 (12.8)	*20.9 (12.7)	31.4 (14.4)	31.4 (16.1)	28.5 (15)	<0.001
V_20_ (%)	16.1 (10.3)	16 (10.3)	5.6 (3.4)	*4.6 (3.3)	7.7 (5.7)	<0.001
V_30_ (%)	11.4 (6.9)	11 (7)	1.5 (1.2)	*0.9 (0.8)	1.5 (1.1)	<0.001
Left lung						
Mean dose (cGy)	2100.4 (250.1)	2085.7 (248.2)	*1345.8 (97.2)	*1356.9 (104.2)	1502.7 (122.1)	<0.001
V_20_ (%)	43.4 (5.7)	43.4 (5.8)	23.4 (2.2)	*20.4 (2.4)	27.8 (3.6)	<0.001
Right lung						
Mean dose (cGy)	99 (33.7)	*97.9 (33.3)	525.8 (148)	614.4 (116.2)	510.8 (97.3)	<0.001
Right breast						
Mean dose (cGy)	147.5 (85.6)	*145.6 (83.4)	555.6 (130.9)	550.1 (157.7)	480.9 (129.8)	<0.001
V_5_ (%)	4.6 (5)	*4.5 (4.9)	43.4 (11.6)	42.3 (17.2)	33.5 (17)	<0.001
D_max_ (cGy)	3214.8 (1251.8)	3171.9 (1202.6)	3026.9 (390.2)	2985.1 (565.6)	2859.5 (479.8)	0.382
Normal tissue						
Mean dose (cGy)	677.1 (126.3)	672.2 (125.9)	655 (108.9)	686.7 (97.9)	680.1 (120.5)	0.081
D_max_ (cGy)	*5634 (316)	5397 (54.2)	5209.6 (94.6)	5212 (106.6)	5252 (50.6)	<0.001

Abbreviations: 3DCRT = three‐dimensional conformal radiotherapy; CTV = clinical target volume; D_95%_ = lowest dose received by at least 95% of the volume, for example, D_98%_ is the lowest dose received by at least 98% of the volume, etc.; D_max_ = Maximum dose; FP‐IMRT = forward‐planned intensity‐modulated radiotherapy; IP‐IMRT = inverse‐planned IMRT; PTV = planning target volume; SD = standard deviation; V_5Gy_ = percentage of the volume receiving 5 Gy or more, for example, V_10Gy_ is the percentage of the volume receiving 10 Gy or more, etc.

* statistically significant values < 0.005.

As a whole, the 9‐field IP‐IMRT technique resulted in more uniform target coverage compared with all other techniques. However, D2% and Dmax doses of IM‐CTV were higher with the 9‐field IP‐IMRT technique than all the other techniques. There was no difference between all the techniques for SC‐CTV. 3DCRT resulted in higher D2% and Dmax doses of axillary‐CTV compared with the other techniques. However, no differences could be observed among the different IMRT techniques.

**Figure 2:**
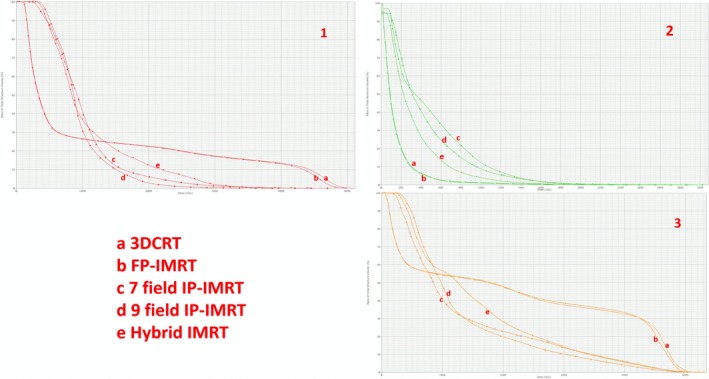
Dose–volume histogram parameters for heart (1), contralateral breast (2), and left lung (3) of five different techniques in an axial image of same patient: (a) three‐dimensional conformal radiotherapy, (b) forward‐planned intensity‐modulated radiotherapy (IMRT), (c) 7‐field inverse‐planned‐IMRT, (d) 9‐field IP‐IMRT, (e) Hybrid IMRT.

When comparing techniques for heart doses, it was observed that the volumes receiving lower doses (V_5Gy_ and V_10Gy_) in FP‐IMRT were significantly lower than in the other techniques (27.4% ± 13.6 and 20.9% ± 12.7, respectively) (Fig. 2). The 9‐field IP‐IMRT, however, achieved the lowest volumes receiving higher doses (V20Gy and V30Gy). However, mean doses did not differ among different techniques.

The right lung sparing was the best with FP‐IMRT (Dmean = 97.9 cGy ± 33.3). For the left lung, 9‐field IP‐IMRT clearly achieved the best sparing (V20Gy = 20.4% ± 2.4). Both 7‐field and 9‐field IP‐IMRT provided a similar mean left lung doses which were smaller than the other techniques (1345.8 cGy ± 97.2 and 1356.9 cGy ± 104.2). There was no difference between the techniques for Dmax doses of right breast. However, FP‐IMRT resulted in lower Dmean and V_5Gy_ compared with the other techniques (Fig. 2‐2). All techniques provided an almost identical Dmean of external normal tissue. However, maximum dose was higher with 3DCRT compared with the other techniques. The hybrid technique resulted in lower doses of right breast in terms of V5Gy, Dmax, and mean doses compared to pure IP‐IMRT techniques (Fig. 2‐2). However, the differences were not statistically significant.

### 3.2 Monitor units

Table 3 summarizes the mean MUs ± standard deviations obtained from each technique. As shown in Table 3, 3DCRT and FP‐IMRT had significantly lower MUs than inverse‐planned IMRT.

**Table 3:** Comparison of MUs between treatment planning techniques.

Technique	Avarage MU ± SD
3DCRT	*420 ± 11
FP‐IMRT	*420 ± 11
7‐field IP‐IMRT	1330 ± 205
9‐field IP‐IMRT	1541 ± 254
*P* values	<0.001

Abbreviations: 3DCRT = three‐dimensional conformal radiotherapy; FP‐IMRT = forward‐planned intensity‐modulated radiotherapy; IP‐IMRT = inverse‐planned IMRT; MU = monitor unit; SD = standard deviation.

* statistically significant values < 0.005.

### 3.3 Results of the TLD measurements

TLD doses calculated in certain points in phantom were compared with the dose calculations obtained from TPS for each technique. The percent difference between TPS and TLD was between 0.6–11.1% for 3DCRT and 0.7–10.9% for FP‐IMRT. The biggest difference was obtained in heart for both techniques. For IP‐IMRT techniques, the percent difference between TPS and TLD was between 1.4 and 7.8% for 7‐field IP‐IMRT and between 1.2 and 9.6% for 9‐field IP‐IMRT techniques.

### 3.4 Results of the skin surface dose measurements

Table 4 summarizes the skin surface doses of 2 Gy per fraction for each technique found by EBT films with no bolus and with 0.5 cm bolus. For 3DCRT and FP‐IMRT techniques, the lateral and central surface doses ranged between 170 ± 2 cGy and 185 ± 4 cGy for 3DCRT, and 172 ± 4 cGy and 179 ± 3 cGy for FP‐IMRT when no bolus was used. Five mm bolus provided 225 ± 4 cGy and 233 ± 3 cGy for 3DCRT, and 220 ± 2 cGy and 227 ± 5 cGy for FP‐IMRT surface doses.

**Table 4:** Comparison of skin doses between treatment planning techniques.

Technique		Medial dose (cGy) ± SD	Central dose (cGy) ± SD	Lateral dose (cGy) ± SD
3DCRT	No bolus	177 ± 5	185 ± 4	170 ± 2
	0.5 cm bolus	225 ± 4	233 ± 3	226 ± 6
FP‐IMRT	No bolus	175 ± 3	179 ± 3	172 ± 4
	0.5 cm bolus	220 ± 2	227 ± 5	225 ± 3
7‐field IP‐IMRT	No bolus	128 ± 6	131 ± 5	122 ± 6
	0.5 cm bolus	206 ± 3	213 ± 4	212 ± 4
9‐field IP‐IMRT	No bolus	132 ± 3	136 ± 2	125 ± 4
	0.5 cm bolus	210 ± 2	216 ± 6	214 ± 5

Abbreviations: 3DCRT = three‐dimensional conformal radiotherapy; FP‐IMRT = forward‐planned intensity‐modulated radiotherapy; IP‐IMRT = inverse‐planned IMRT; SD = standard deviation.

For IP‐IMRT, these figures were in the range of 125 ± 4 cGy to 136 ± 2 cGy when there was no bolus. Five mm bolus caused a more homogeneous and effective surface doses ranging between 206 ± 3 cGy and 216 ± 6 cGy.

## 4 DISCUSSION

In this study, we compared five different techniques 3DCRT, FP‐IMRT, 7‐ and 9‐field IP‐IMRT, and hybrid IMRT in order to find the optimal technique in ten patients with left‐sided breast carcinoma with intent to treat the CW and entire lymphatics. We found similar PTV coverage both in chest wall and lymphatics in all techniques except forward planning IMRT in which the D95% of chest wall PTV was slightly but significantly lower than the other techniques. However, when evaluating the Dmax and D2% of chest wall CTV, the dose distribution of 3DCRT technique revealed significantly higher doses compared to the other techniques. The dose homogeneity in chest wall CTV was better in IMRT techniques compared to the 3DCRT. Again IMRT especially 9‐field IP‐IMRT provided more homogeneous dose distribution in internal mammary and axillary CTVs. In literature, the partially wide tangential field technique is regarded as the best 3DCRT technique for breast/CW and full lymphatic irradiation.^14^, ^15^, ^16^ However, in recent years with the introduction of modern treatment machines, FP‐IMRT using FINF technique is accepted as standard technique in breast carcinoma radiatıon therapy. IP‐IMRT has been shown to increase the dose homogeneity in CTVs, decrease the high‐dose regions in OARs while increasing the low‐dose areas in normal tissues.^17^ The most widely used technique in IP‐IMRT is with either seven or nine fields.

In our planning and dosimetric study, we found similar PTV coverage both in CW and lymphatics in all techniques except FP‐IMRT in which the D95% of CW‐PTV was slightly but significantly lower than the other techniques. However, when evaluating the Dmax and D2% of CW‐CTV, the dose distribution of 3DCRT technique revealed significantly higher doses compared to the other techniques. The dose homogeneity in CW‐CTV was better in IMRT techniques compared to the 3DCRT. Again IMRT, especially 9‐field IP‐IMRT provided more homogeneous dose distribution in IM and axillary CTVs.

The improvement of target coverage of chest wall and local lymphatics with IP‐IMRT has been shown by several studies.^18^ In a study by Dogan et al., 6‐ and 9‐field IP‐IMRT were shown to provide much better coverage compared to the 2‐ and 4‐field IP‐IMRT techniques.^5^ In this particular study, the largest difference in average D95% of the internal mammary PTV between 3DCRT and IMRT was reported to be with 9‐field IP‐IMRT which is similar to ours.

Both FP‐IMRT and IP‐IMRT techniques in our study resulted in similar CW and regional nodal coverage. In the FP‐IMRT technique, we prescribed the 5–10% of the dose with segmental blocking of the hot volumes receiving ˃107% of the dose or heart and lung tissue and produced more homogeneous dose coverage compared with 3DCRT without any difference from IP‐IMRT techniques. This FP‐IMRT technique provided us to spare the contralateral breast and deliver the dose within shorter treatment times. Jagsi et al. compared 9‐field IP‐IMRT, tangential beamlet technique with three to five ipsilateral beams, a segmental technique using FP‐multisegments and a FP‐segmental blocked technique to limit heart dose and found that the primary differences between the techniques were only in OARs.^18^ However, in a recent dosimetric study by Ma et al., the IP‐IMRT techniques as 5‐field IMRT and 2‐field‐VMAT plans, which showed similar PTV coverage, and conformity exhibited higher PTV coverage compared to 3DCRT‐FINF technique.^19^

Long‐term cardiac effects are an important component of survivorship after breast cancer radiotherapy. In a study by Darby et al. which included patients treated between 1958 and 2001, it was shown that every 1 Gy increase in mean heart dose caused a relative risk increase of cardiac events by 7%.^20^ The pathophysiology of radiation‐induced cardiac disease is shown by the radiation effect on the vasculature, pericard, and myocardium and a lesser extent on the valvular tissue.^21^ Numerous studies have shown that the injury of endothelial cells were the major component of vascular injury.^22^, ^23^ In our previous experimental electron microscopy study, we have shown that deleterious effects of radiation on vasculature were prominent especially when high dose was applied.^24^ The long‐term clinical data regarding the cardiotoxic effects of radiotherapy mostly come from the long‐term survivors of lymphoma patients. In a recent case–control study by Nimwegen et al., the risk of coronary heart disease increased linearly with increasing mean heart dose with a median interval between radiotherapy and heart disease of 19 yr.^25^ In that particular study, there was a 2.5‐fold increase in risk of coronary heart disease for patients receiving a mean heart dose of 20 Gy when compared to patients not treated with mediastinal radiotherapy. In our dosimetric study, when compared all the techniques for heart doses, it was observed that the volumes receiving lower doses (V_5Gy_ and V_10Gy_) in forward‐planned IMRT were significantly lower than the other techniques which were 27.4% ± 13.6 and 20.9% ± 12.7, respectively. However, with the IP‐IMRT techniques, volumes receiving high doses as V_20_ and V_30_ were significantly lower than 3DCRT and FP‐IMRT techniques. The 9‐field IMRT, however, achieved the lowest volumes receiving higher doses.

There are several techniques regarding the cardiac avoidance in breast radiotherapy. The most frequently used techniques for cardiac avoidance are deep inspiration breath hold (DIBH) or inspiratory gating techniques. In one study with the use of DIBH technique, the median heart volume receiving >50% of the dose was decreased from 19% to <3%.^26^ In our study, we used only free breathing technique for treatment planning and we think that the weakest part of our study is not using breath hold technique in addition to the other techniques.

A strong relationship between radiation exposure and cancer has been shown especially by epidemiological studies conducted for survivors of atomic bombings in Japan as well as studies for medically exposed patients or radiation workers.^27^, ^28^ The overall risk of fatal cancers is estimated to be 8%**/**1 Gy.^29^ In a study by Zhang et al. among 5248 patients treated for breast cancer between 1965 and 1994, there was an increased risk of all second cancers combined following radiotherapy with a relative risk of 1.22.^30^ The increased risk in that particular study was apparent 5 or more years after radiotherapy. In addition, it has been estimated that the conversion of treatment plan from 3D conformal radiotherapy to IMRT, due to the increase of volumes receiving lower doses, will cause additional 0.5% of surviving patients with second malignancy.^29^ Moreover, an additional 0.25% of surviving patients will develop a radiation‐induced malignancy because of the increase in monitor units in IMRT. As expected, 3DCRT and forward‐planned IMRT had significantly lower MUs than inverse‐planned IMRT in our study. The mean MU was 420 in 3DCRT and forward‐planned IMRT, whereas it was calculated as 1330 MU ± 205 for 7‐field inverse‐planned IMRT, and 1541 ± 254 for 9‐field inverse‐planned IMRT which may lead to increase in radiation‐induced secondary malignancy in long‐term follow‐up. Moreover, V5Gy in the contralateral breast was significantly less in techniques using either 3D‐CRT or field‐in‐field technique. There was decrease of V5Gy with hybrid technique, but this decrease was small. Since it is a dosimetric study that did not aim to focus on secondary malignancies, we cannot estimate the risk of radiation‐induced malignancies.

## 5 CONCLUSION

In left chest wall and lymphatic irradiation, IP‐IMRT techniques provide more homogeneous dose coverage when compared to 3DCRT and produce significant sparing in the heart tissue and left lung. Forward‐planned IMRT has an advantage to achieve significantly lower dose to the contralateral breast tissue when compared to IP‐IMRT. The hybrid technique seems to combine the advantages of both techniques and be promising. The secondary malignancy risk and heart complications should be kept in mind when treatment plans are made.

## CONFLICT OF INTEREST

No conflict of interest.
